# Speech decoding using cortical and subcortical electrophysiological signals

**DOI:** 10.3389/fnins.2024.1345308

**Published:** 2024-02-29

**Authors:** Hemmings Wu, Chengwei Cai, Wenjie Ming, Wangyu Chen, Zhoule Zhu, Chen Feng, Hongjie Jiang, Zhe Zheng, Mohamad Sawan, Ting Wang, Junming Zhu

**Affiliations:** ^1^Department of Neurosurgery, Second Affiliated Hospital, Zhejiang University School of Medicine, Hangzhou, China; ^2^Clinical Research Center for Neurological Disease of Zhejiang Province, Hangzhou, China; ^3^Department of Neurology, Epilepsy Center, Second Affiliated Hospital, Zhejiang University School of Medicine, Hangzhou, China; ^4^CenBRAIN Lab, School of Engineering, Westlake University, Hangzhou, China; ^5^School of Foreign Languages, Tongji University, Shanghai, China; ^6^Center for Speech and Language Processing, Tongji University, Shanghai, China

**Keywords:** speech, decoding, sEEG, machine learning, neural network

## Abstract

**Introduction:**

Language impairments often result from severe neurological disorders, driving the development of neural prosthetics utilizing electrophysiological signals to restore comprehensible language. Previous decoding efforts primarily focused on signals from the cerebral cortex, neglecting subcortical brain structures’ potential contributions to speech decoding in brain-computer interfaces.

**Methods:**

In this study, stereotactic electroencephalography (sEEG) was employed to investigate subcortical structures’ role in speech decoding. Two native Mandarin Chinese speakers, undergoing sEEG implantation for epilepsy treatment, participated. Participants read Chinese text, with 1–30, 30–70, and 70–150 Hz frequency band powers of sEEG signals extracted as key features. A deep learning model based on long short-term memory assessed the contribution of different brain structures to speech decoding, predicting consonant articulatory place, manner, and tone within single syllable.

**Results:**

Cortical signals excelled in articulatory place prediction (86.5% accuracy), while cortical and subcortical signals performed similarly for articulatory manner (51.5% vs. 51.7% accuracy). Subcortical signals provided superior tone prediction (58.3% accuracy). The superior temporal gyrus was consistently relevant in speech decoding for consonants and tone. Combining cortical and subcortical inputs yielded the highest prediction accuracy, especially for tone.

**Discussion:**

This study underscores the essential roles of both cortical and subcortical structures in different aspects of speech decoding.

## Introduction

Humans use a complex process to speak, involving rapid planning of phonemes (sound units) in words, engaging prefrontal brain regions within a larger language network responsible for word and sentence formation (Bohland and Guenther, 2006; Fedorenko et al., 2016; Kazanina et al., 2018; Hoffman, 2019). This network is connected to areas controlling their physical production (Duffau et al., 2003; Ikeda et al., 2014). Studies using cortical surface recordings have found that phonetic features are organized in specific regions and can be decoded from brain activity in posterior prefrontal and premotor areas, indicating a structured cortical organization (Anumanchipalli et al., 2019; Wang et al., 2023). Despite advancements, fully understanding the mechanism of speech planning and production remains a challenge.

Recently, there has been a significant interest in Brain-Computer Interfaces (BCIs) that can interpret speech from brain signals, potentially aiding those unable to speak (Metzger et al., 2023; Willett et al., 2023). Understanding the mechanism of speech generation in the brain, including the sequence and location of involved brain regions, is crucial for developing a speech neuroprosthesis.

Current methods can decode text representations from neural signals during actual speech generation, spanning phonemes, words, full sentences, and even keywords. Many of these advancements utilize neural signals from cortical regions, including electrocorticography (ECoG) and Utah array, to record neural activity with high precision in time and space. While there are models explaining speech generation, the exact involvement of all brain regions remains unclear. Research now suggests that deeper brain areas like the hippocampus and thalamus play a role in both language comprehension and speech generation.

Stereotactic EEG (sEEG) is another commonly used surgical technique to record intracranial neurophysiological signals, where electrodes are implanted through small openings in the skull for treatment of refractory epilepsy. Unlike ECoG, which only records in cortical regions, sEEG is able to sample various regions, including subcortical brain structures, potentially benefiting BCI applications utilizing distant and deep brain areas.

Here, we hypothesize that neural signals from subcortical brain regions can contribute to speech decoding. To validate our hypothesis that subcortical brain regions contribute to speech decoding, we asked participants to vocalize all possible pronunciation of characters in Mandarin Chinese while both their voices and sEEG data were recorded.

## Materials and methods

**Figure 1:**
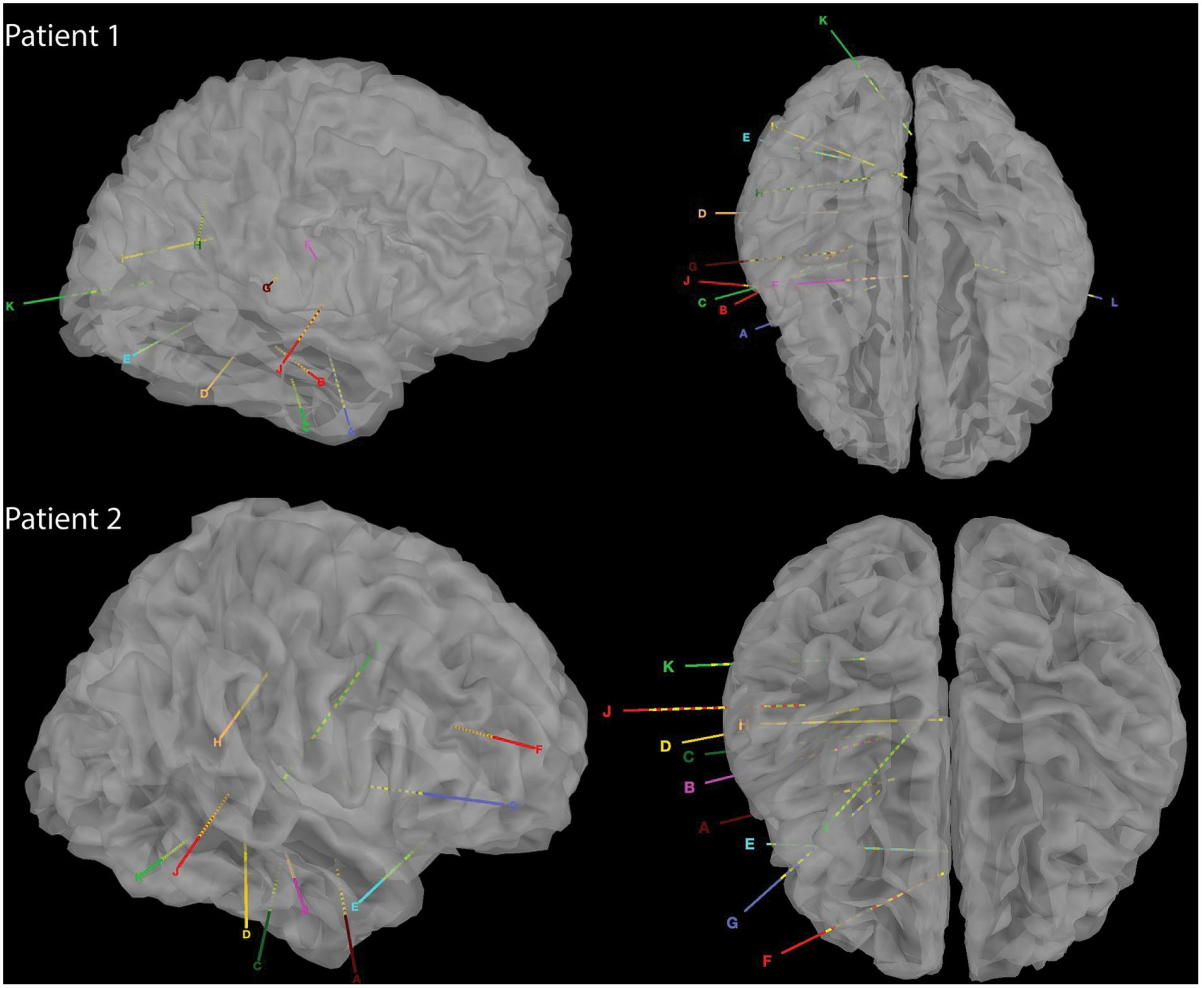
Reconstructed CT images of sEEG implants in the two patients.

Two native Mandarin Chinese speaking patients with refractory epilepsy underwent sEEG surgeries. Patient 1 had a history of refractory epilepsy (generalized tonic–clonic seizure) of 25 years; patient 2 had a history of refractory epilepsy (absence seizure) of 11 years. No abnormality was reported during neuropsychological testing. WADA test showed that the left hemisphere is the language-dominant hemisphere in both patients. To localize seizure foci, sEEG electrodes (0.8 mm diameter, 2 mm contact length with 1.5 mm intercontact distance; Sinovation (Beijing) Medical Technology Co., Ltd.) were implanted in cortical structures including superior temporal gyrus, middle temporal gyrus, and inferior temporal gyrus, and subcortical structures, including thalamus (ventral nuclear group, including ventroanterior and ventrolateral nuclei), hippocampus, insular gyrus, parahippocampal gyrus, and amygdala (although the parahippocampal gyrus and anterior cingulate cortex are archipaleocortex and paleocortex, both structures are situated beneath the neocortex, rendering them inaccessible to surface ECoG electrodes. Consequently, for the purposes of facilitating comparisons, they are designated as subcortical regions in this study; Figure 1). The positions of the electrodes were confirmed manually by merging postoperative CT with preoperative MR (Supplementary material). As the majority of the electrodes were located in the right hemisphere, electrodes in the left hemisphere were not included in this study. This clinical trial was approved by the Ethics Committee of the Zhejiang University School of Medicine Second Affiliated Hospital (protocol number: I2022145).

**Figure 2:**
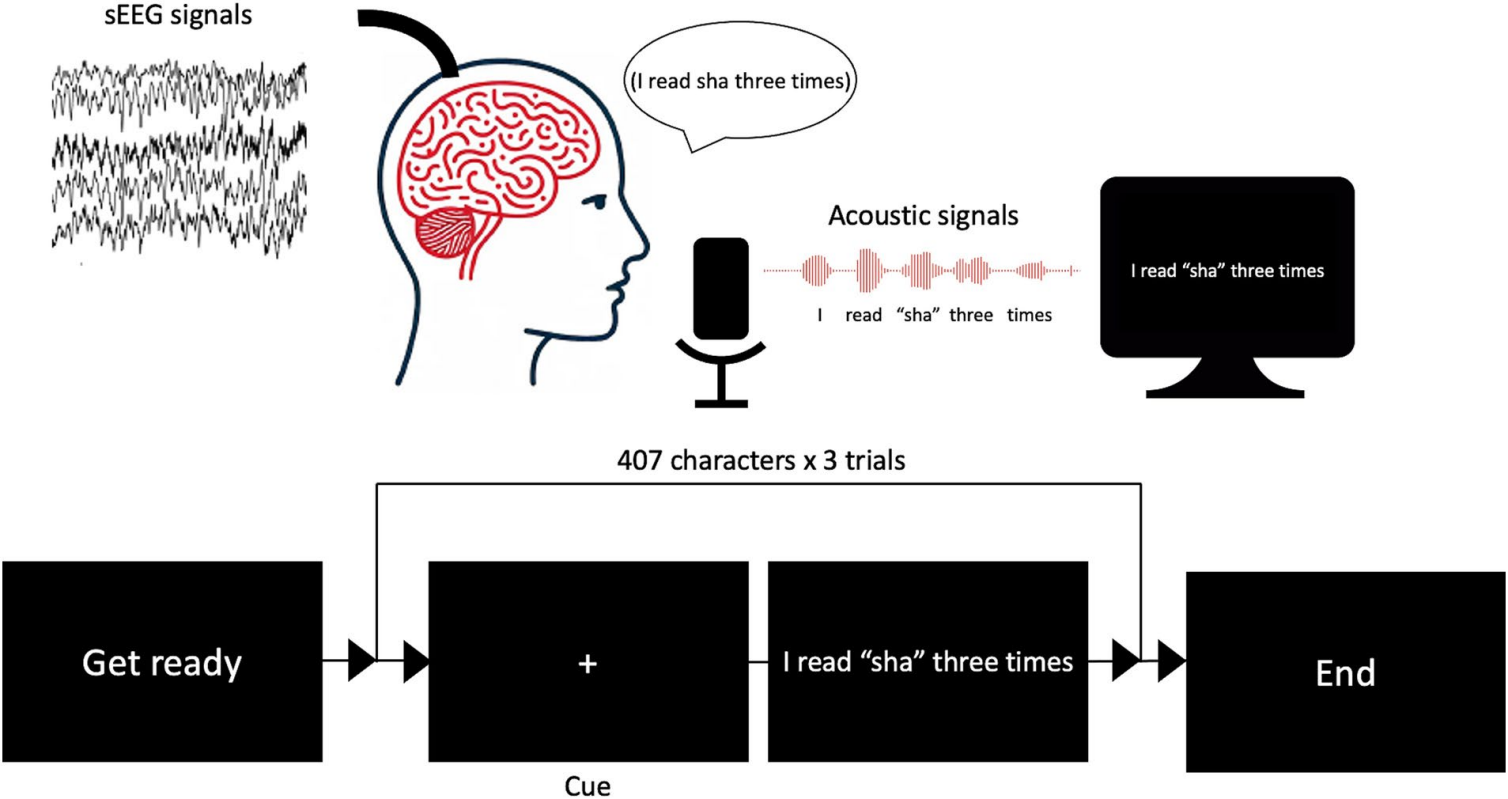
Schematic diagram of experimental design to record vocal and electrophysiological signals simultaneously.

During the 2-week window to localize seizure foci, we asked the patients to speak out loud when a cue was given while simultaneously recording their voice and synchronized intracranial neurophysiological signals (Figure 2). A total of 407 characters were recorded over repeated trials, covering all possible pronunciations and tones in Mandarin Chinese (Supplementary Table S1).

### sEEG and acoustic signal processing

A total of 290 (148 + 142) sEEG contacts were implanted, sampled at 2 k Hz (Nihon Kohden Corp). We began signal processing by linearly detrending the sEEG signals and performed anti-aliasing low-pass filtering at 500 Hz. For extracting valuable insights from the sEEG signals, we determined the power in the 1–30 Hz, 30–70 Hz, and 70–150 Hz frequency range, which is believed to represent ensemble spiking and offers specific data about movement and speech functions. The amplitude of the 70–150 Hz frequency component was extracted with the Hilbert transform and down-sampled to 200 Hz. The 1–30 Hz and 30–70 Hz frequency components were extracted with a 6th order Butterworth bandpass filter, also down-sampled to 200 Hz and parallelly aligned with the 70–150 Hz amplitude. Then the signals were z-scored using a 30 s window of running mean and standard deviation to normalize data distribution (each contact’s activity was normalized to have zero mean and a variance of one). Regarding the acoustic data, voicing of each character was semi-automatically segmented, and categorized based on vowels, consonants, and tuning. Each consonant can be assigned a corresponding set of articulatory places and manners based on international standard (Table 1) (Yonghong Li et al., 2015). The articulatory place is the location within the mouth where a speech sound is made. In English, there are ten places of articulation for consonants: bilabial, labiodental, dental, alveolar, post-alveolar, palato-alveolar, palatal, velar, glottal, and retroflex. In Mandarin Chinese, there are 7 articulatory places, which include: bilabials, labiodentals, dentals, alveolars, post-alveolars, palatals and velars. The articulatory manner of a sound is how the airstream is affected as is goes through vocal tract. In Mandarin Chinese, there are 8 articulatory manners, which include: plosives (unaspirated and aspirated), affricates (unaspirated and aspirated), fricatives (voiceless and voiced), nasals and laterals. Power features of synchronized sEEG signals were segmented and categorized accordingly.

**Table 1:** Place-voice-manner consonant chart for Mandarin Chinese.

Articulatory place	Plosive		Affricate		Fricative		Nasal	Lateral
	Unaspirated	Aspirated	Unaspirated	Aspirated	Voiceless	Voiced	Voiced	Voiced
Bilabial	b[p]	p[p^ɦ^]					m[m]	
Labiodental					f[f]			
Dental			z[ts]	c[ts^ɦ^]	s[s]			
Alveolar	d[t]	t[t^ɦ^]					n[n]	l[l]
Post-alveolar			zh[tʂ]	ch[tʂ^ɦ^]	sh[ʂ]	r[ʐ]		
Palatal			j[tɕ]	q[tɕ^ɦ^]	x[ɕ]			
Velar	g[k]	k[k^ɦ^]			h[x]			

### Speech decoding from sEEG signals using recurrent neural network

We used a stacked 3-layer bidirectional long short-term memory (bLSTM; 100 hidden units for each cell) recurrent neural network to decode articulatory features (articulatory places, manners, and tuning) from continuous neural activity (1–30 Hz, 30–70 Hz, and 70–150 Hz components). The model learned the mapping between 200 ms sequences of 1–30 Hz, 30–70 Hz, and 70–150 Hz components and a corresponding single time point (sampled at 200 Hz) of the articulatory features. During testing, a full pronunciation a character of neural activity was processed by the decoder, which processed 200 ms of data at a time, sliding over the sequence sample by sample, until it has returned a sequence of articulatory features that is equal length to the neural data. The neural data was padded with an additional 100 ms of data before and after the sequence to ensure the result was the correct length. The model was trained using the Adam optimizer to minimize mean-squared error (initialized with learning rate = 0.001, beta1 = 0.9, beta2 = 0.999, epsilon = 1e-8). Models were stopped from training after the validation loss no longer decreased. Dropout rate was set to 50%. Training and testing data (325.82 ratio) were randomly split based off of recording sessions (training and test sets collected from different recording sessions; repeated 1,000 times). Data was shuffled to the order of the electrodes that were fed into the decoder. Models were coded using Python’s version 1.9 of Tensorflow.

## Results

### Decoding consonants based on articulatory place and articulatory manner classification using sEEG signals from single region

**Figure 3:**
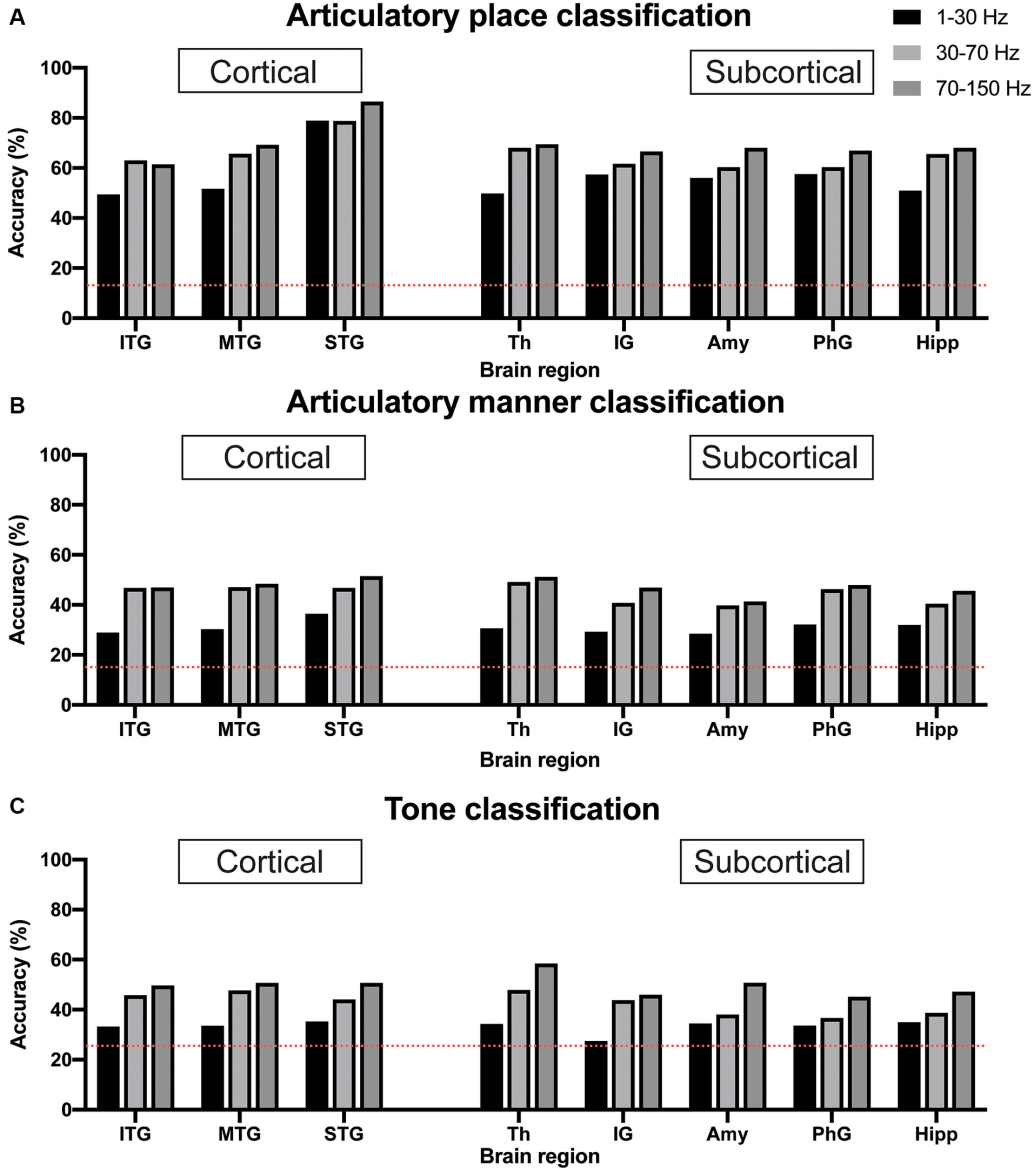
Classification accuracy for articulatory place **(A)**, articulatory manner **(B)**, and tone **(C)** using electrophysiological signals from cortical vs. subcortical brain regions. Dotted red lines indicate chance levels. **(A)** sEEG features from the superior temporal gyrus generate the best prediction results for articulatory place. **(B)** sEEG features from the superior temporal gyrus and the thalamus generate the best prediction results at similar levels for articulatory manner. **(C)** sEEG features from the thalamus generate the best prediction results for tone. Power in the 70–150 Hz frequency band is best feature for prediction vs. powers in the 1–30 Hz and 30–70 Hz frequency bands. ITP, inferior temporal gyrus; MTP, middle temporal gyrus; STP, superior temporal gyrus; Th, thalamus; IG, insular gyrus; Amy, amygdala; PhG, parahippocampal gyrus; Hipp, hippocampus.

We used sEEG 1–30 Hz, 30–70 Hz, and 70–150 Hz frequency band power of electrophysiological signals from individual brain regions to classify articulatory place and articulatory manner. The pure chance level for articulatory place and articulatory manner classification was 0.143 (1/7) and 0.125 (1/8), respectively. Our results indicated that 70–150 Hz frequency band power showed the best classification capability for both articulatory place and manner prediction across brain regions in general, which was in line with previous reports (Moses et al., 2021). For articulatory place classification, the superior temporal gyrus showed the best performance, with an accuracy of 86.5% (Figure 3A). For articulatory manner classification, the superior temporal gyrus and the thalamus had the best results, classifying successfully 51.5 and 51.7% of the articulatory manner, respectively (Figure 3B).

### Decoding tones using sEEG signals from single region

Similar to articulatory place and manner decoding, we used 1–30 Hz, 30–70 Hz, and 70–150 Hz frequency band power of sEEG electrophysiological signals from individual brain regions to classify tone. The pure chance level for tone classification was 0.25 (1/4). Our results indicated that 70–150 Hz frequency band power still possessed the best classification capability for tone prediction across brain regions in general, and the thalamus showed the best performance, with an accuracy of 58.3% (Figure 3C).

### Decoding consonants and tones using sEEG signals from cortical and subcortical regions combined

**Figure 4:**
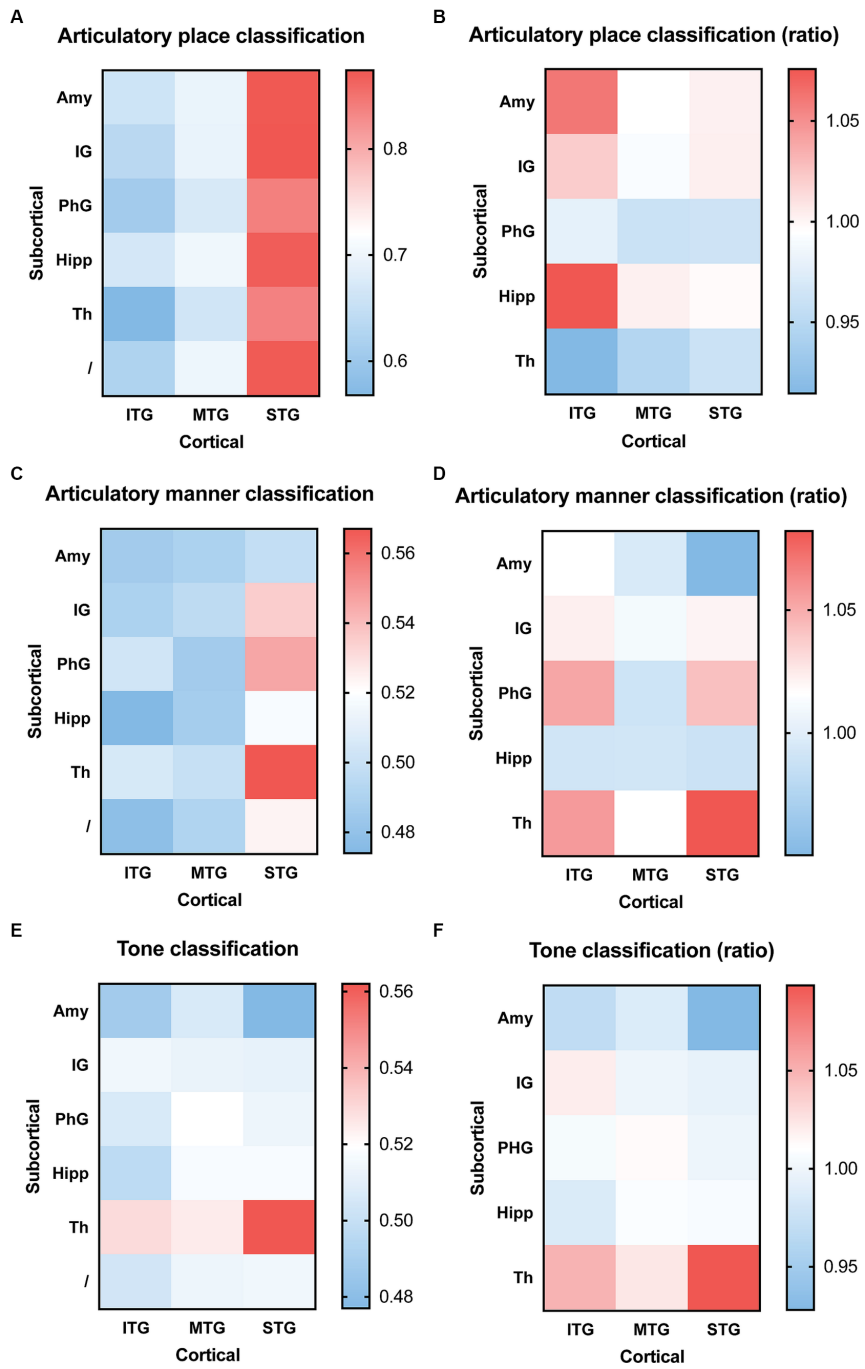
Classification accuracy and improvement ratio for articulatory place **(A,B)**, articulatory manner **(C,D)**, and tone **(E,F)** when cortical electrophysiological signals were combined with subcortical electrophysiological signals. **(A,B)** sEEG features from the superior temporal gyrus are best at predicting articulatory place, with or without sEEG input from subcortical regions. sEEG features from the inferior temporal gyrus may benefit from sEEG input from subcortical regions during articulatory place prediction, but its absolute accuracy remains lower than sEEG features from the superior temporal gyrus alone. **(C,D)** sEEG features from the superior temporal gyrus combined with sEEG features from the thalamus produce the best prediction results for articulatory manner, higher than the prediction accuracy generated from sEEG features from these two structures alone. **(E,F)** sEEG features from the superior temporal gyrus combined with sEEG features from the thalamus produce the best prediction results for tone, but it remains lower than the prediction accuracy generated from sEEG features from the thalamus alone. ITP, inferior temporal gyrus; MTP, middle temporal gyrus; STP, superior temporal gyrus; Th, thalamus; IG, insular gyrus; Amy, amygdala; PhG, parahippocampal gyrus; Hipp, hippocampus.

We then used combined electrophysiological signals, one channel from cortical and one channel from subcortical brain regions, to decode consonants and tones. For articulatory place classification, we found that sEEG signals from the superior temporal gyrus were able to produce best classification results, with or without sEEG signals from subcortical regions (Figure 4A). Combining input signals from inferior temporal gyrus with hippocampus improved prediction, but still lower than what superior temporal gyrus was able to predict by itself (Figure 4B). For articulatory manner classification, sEEG signals from the superior temporal gyrus combined with signals from the thalamus were able to make best prediction (Figures 4C,D). For tone classification, sEEG signals from the thalamus profoundly improved classification results when combined with signals from the inferior, middle, and superior temporal gyri, still producing the best results when combined with the superior temporal gyrus (Figures 4E,F).

## Discussion

Our work demonstrates the feasibility and value of electrophysiological signals recorded in both cortical and subcortical regions for speech decoding. Our findings are particularly significant for the design of speech neuroprostheses, as they suggest that incorporating signals from both cortical and subcortical structures could enhance the performance of these devices. The center for language processing is generally believed to be in the cortical area around the sylvian fissure of the left hemisphere called the perisylvian area. Past studies focus on harvesting signals from this area for speech decoding, while other studies have indicated the involvement of subcortical structures, such as the hippocampus and the thalamus, during speech processing (Duff and Brown-Schmidt, 2012; Hebb and Ojemann, 2013; Klostermann et al., 2013; Covington and Duff, 2016; Piai et al., 2016).

In our study, the perisylvian area, i.e., superior temporal gyrus remains highly relevant for speech decoding. We are able to use signals from the superior temporal gyrus to classify articulatory place and articulatory manner, which will help predict consonants, as well as tone classification. Signals from subcortical areas seem less relevant for articulatory place prediction, when superior temporal gyrus is used. But for articulatory area and tone predictions, signals from the thalamus substantially improve accuracy when combined with signals from the superior temporal gyrus. Interestingly, the prediction accuracy for articulatory place is the highest, while its chance level is the lowest, compared to articulatory manner and tone. We do not have a clear explanation for this, but we believe it reflects the neural representation of the signals captured. Another interesting finding is that thalamic neural signals are best for tone prediction, which may serve as an important piece of information for research in the field of evolutionary linguistics.

Currently there are several groups investigating the use of sEEG signals for speech decoding. Angrick et al. (2021) show that sEEG and cortical-only ECoG yield similar results for speech decoding. Soroush et al. (2022) study signals from grey and white matter for speech activity detection. The same group also report significant contributions from deep brain structures for speech decoding (Soroush et al., 2023). Thomas et al. (2023) use sEEG approach but only include cortical regions in their study, and report neural correlates in multiple cortical regions for both articulatory and phonetic components. Ramos-Escobar et al. (2022) report evidence of hippocampal involvement in the speech segmentation process. Cometa et al. (2023) discovered involvement from both cortical and subcortical in syntactic processing, including from the non-dominant hemisphere. Verwoert et al. (2022) published an open access sEEG dataset of 10 participants reading Dutch words. Afif et al. (2010) also reported speech arrest after stimulating the insula electrically, implicating speech production in subcortical areas.

There are also studies using noninvasive modalities [electroencephalogram (EEG) or magnetoencephalography (MEG)] to investigate their value for speech decoding. Sereshkeh et al. focused on decoding yes/no responses to binary questions using EEG. They utilized a 64-channel EEG system and applied a regularized neural network for classification, achieving notable accuracy (Sereshkeh et al., 2017). Min et al. used vowel sounds as EEG prompts and employed sparse regression models for feature selection, along with extreme learning machines (ELM) for classification. This approach yielded significant results in classifying the vowel-based imagined speech (Min et al., 2016). Nguyen et al. introduced a novel approach using channel cross-covariance matrices in Riemannian manifold for feature representation. They demonstrated improved classification accuracy by combining temporal and wavelet domain features (Nguyen et al., 2018). Jahangiri and Sepulveda focused on classifying four phonetically dissimilar syllables using EEG. They utilized Gabor wavelets for feature extraction and achieved significant differentiation between the syllables (Jahangiri and Sepulveda, 2018). Koizumi et al. involved the use of Japanese words as prompts and extracted band powers from EEG channels. They reported higher classification accuracy, particularly when using features extracted from the high gamma band (Koizumi et al., 2018). Dash et al. reported decoding results from spoken and imaged phrases using MEG signals. They found that CNNs were highly effective decoders, with an average decoding accuracy of up to 93% for the imagined and 96% for the spoken phrases (Dash et al., 2020). Beach et al. found that the neural representation of isolated speech sounds includes concurrent phonemic and subphonemic information. This was determined through their study using MEG during tasks that required participants to either passively listen to or actively categorize speech sounds. The study revealed that linear classifiers could decode the perception of different speech sounds, and the categorization process did not require the loss of subphonemic detail (Beach et al., 2021).

Our study has limitations. The sample size, comprising only two Mandarin Chinese-speaking individuals, limits the generalizability of our findings. Additionally, the study’s focus on right hemisphere regions could miss critical information processed in the left hemisphere, traditionally associated with language. Furthermore, the clinical condition of our participants (refractory epilepsy) and the resulting altered neurophysiology could affect the generalizability of our findings to the broader population.

Looking forward, our research opens several avenues for further investigation. Larger-scale studies involving diverse languages and larger participant cohorts could validate and extend our findings. Moreover, longitudinal studies could examine the stability of sEEG signal decoding over time, which is crucial for the practical application of BCIs in chronic conditions. Finally, integrating our findings with machine learning advancements could lead to more sophisticated and accurate speech neuroprosthesis designs, ultimately enhancing the quality of life for individuals with speech impairments.

In conclusion, our study represents a significant step towards understanding and harnessing the full potential of brain signals for speech decoding. The implications for assistive technologies are profound, offering a chance for restoring communication abilities to those who have lost them.

## Data availability statement

The raw data supporting the conclusions of this article will be made available by the authors, without undue reservation.

## Ethics statement

The studies involving humans were approved by Zhejiang University School of Medicine Second Affiliated Hospital. The studies were conducted in accordance with the local legislation and institutional requirements. The participants provided their written informed consent to participate in this study.

## Author contributions

HW: Conceptualization, Data curation, Formal analysis, Funding acquisition, Investigation, Methodology, Project administration, Resources, Supervision, Validation, Visualization, Writing – original draft, Writing – review & editing. CC: Validation, Visualization, Writing – review & editing. WM: Data curation, Investigation, Supervision, Writing – review & editing. WC: Funding acquisition, Visualization, Writing – review & editing. ZhoZ: Conceptualization, Methodology, Writing – review & editing. CF: Data curation, Methodology, Writing – review & editing. HJ: Data curation, Writing – review & editing. ZheZ: Data curation, Methodology, Writing – review & editing. MS: Supervision, Validation, Writing – review & editing. TW: Conceptualization, Methodology, Supervision, Validation, Writing – review & editing. JZ: Funding acquisition, Project administration, Supervision, Validation, Writing – review & editing.
