# Supplementary material for: Speech decoding using cortical and subcortical electrophysiological signals
Source: Front Neurosci. 2024 Feb 29;18:1345308. doi: 10.3389/fnins.2024.1345308 (PMC10937352; doi:10.3389/fnins.2024.1345308)
Supplement: Supplementary file 1 [file Table_1.PDF]

Articulatory place	Plosive		Affricate		Fricative		Nasal	Lateral
	**unaspirated**	**aspirated**	**unaspirated**	**aspirated**	**voiceless**	**voiced**	**voiced**	**voiced**
**Bilabial**	b[p]	p[p^{ɦ}]					m[m]	
**Labiodental**					f[f]			
**Dental**			z[ts]	c[ts^{ɦ}]	s[s]			
**Alveolar**	d[t]	t[t^{ɦ}]					n[n]	l[l]
**Post-alveolar**			zh[tʂ]	ch[tʂ^{ɦ}]	sh[ʂ]	r[ʐ]		
**Palatal**			j[tɕ]	q[tɕ^{ɦ}]	x[ɕ]			
**Velar**	g[k]	k[k^{ɦ}]			h[x]			
