# Supplementary material for: Speech decoding using cortical and subcortical electrophysiological signals
Source: Front Neurosci. 2024 Feb 29;18:1345308. doi: 10.3389/fnins.2024.1345308 (PMC10937352; doi:10.3389/fnins.2024.1345308)
Supplement: Supplementary file 2 [file Table_2.PDF]

	a	o	e	i	-i	u	ü	ai	ei	ui	ao	ou	iu	ie	üe	er	an	en	in	un	ün	ang	eng	ing	ong	i-a	i-an	i-ang	i-ao	i-ong	u-a	u-ai	u-an	u-ang	u-o	ü-an
b	ba	bo		bi		bu		bai	bei		bao			bie			ban	ben	bin			bang	beng	bing			bian		biao							
p	pa	po		pi		pu		pai	pei		pao	pou		pie			pan	pen	pin			pang	peng	ping			pian		piao							
m	ma	mo	me	mi		mu		mai	mei		mao	mou	miu	mie			man	men	min			mang	meng	ming			mian		miao							
f	fa	fo				fu			fei			fou					fan	fen				fang	feng													
d	da		de	di		du		dai	dei	dui	dao	dou	diu	die			dan	den		dun		dang	deng	ding	dong	dia	dian		diao				duan		duo	
t	ta		te	ti		tu		tai		tui	tao	tou		tie			tan			tun		tang	teng	ting	tong		tian		tiao				tuan		tuo	
n	na		ne	ni		nu	nü	nai	nei		nao	nou	niu	nie	nüe		nan	nen	nin			nang	neng	ning	nong		nian	niang	niao				nuan		nuo	
l	la	lo	le	li		lu	lü	lai	lei		lao	lou	liu	lie	lüe		lan		lin	lun		lang	leng	ling	long	lia	lian	liang	liao				luan		luo	
g	ga		ge			gu		gai	gei	gui	gao	gou					gan	gen		gun		gang	geng		gong						gua	guai	guan	guang	guo	
k	ka		ka			ku		kai	kei	kui	kao	kou					kan	ken		kun		kang	keng		kong						kua	kuai	kuan	kuang	kuo	
h	ha		he			hu		hai	hei	hui	hao	hou					han	hen		hun		hang	heng		hong						hua	huai	huan	huang	huo	
j				ji			ju						jiu	jie	jue				jin		jun			jing		jia	jian	jiang	jiao	jiong						juan
q				qi			qu						qiu	qie	que				qin		qun			qing		qia	qian	qiang	qiao	qiong						quan
x				xi			xu						xiu	xie	xue				xin		xun			xing		xia	xian	xiang	xiao	xiong						xuan
zh	zha		zhe		(zhi)	zhu		zhai	zhei	zhui	zhao	zhou					zhan	zhen		zhun		zhang	zheng		zhong						zhua	zhuai	zhuan	zhuang	zhuo	
ch	cha		che		(chi)	chu		chai		chui	chao	chou					chan	chen		chun		chang	cheng		chong						chua	chuai	chuan	chuang	chuo	
sh	sha		she		(shi)	shu		shai	shei	shui	shao	shou					shan	shen		shun		shang	sheng								shua	shuai	shuan	shuang	shuo	
r			re		(ri)	ru				rui	rao	rou					ran	ren		run		rang	reng		rong								ruan		ruo	
z	za		ze		(zi)	zu		zai	zei	zui	zao	zou					zan	zen		zun		zang	zeng		zong								zuan		zuo	
c	ca		ce		(ci)	cu		cai		cui	cao	cou					can	cen		cun		cang	ceng		cong								cuan		cuo	
s	sa		se		(si)	su		sai		sui	sao	sou					san	sen		sun		sang	seng		song								suan		suo	
y	ya	yo			(yi)		(yu)				yao	you		(ye)	(yue)		yan		(yin)		(yun)	yang		(ying)	yong											(yuan)
w	wa	wo				(wu)		wai	wei								wan	wen				wang	weng													
