# Supplementary figures and images for: Speech decoding using cortical and subcortical electrophysiological signals
Source: Front Neurosci. 2024 Feb 29;18:1345308. doi: 10.3389/fnins.2024.1345308 (PMC10937352; doi:10.3389/fnins.2024.1345308)

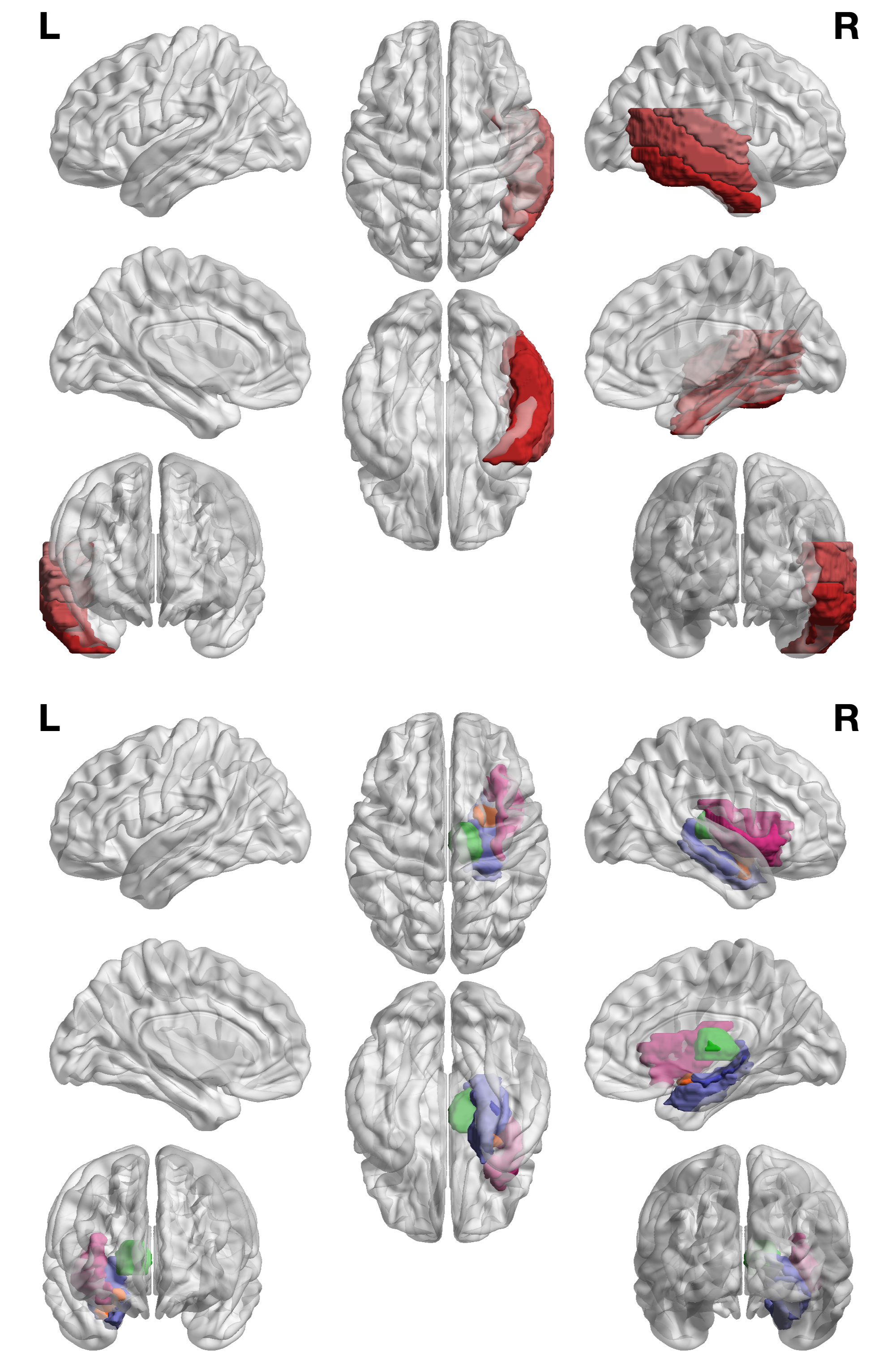

Supplement: Supplementary file 4 [file Image_1.TIF]
